# On-Chip Facile Preparation of Monodisperse Resorcinol Formaldehyde (RF) Resin Microspheres

**DOI:** 10.3390/mi9010024

**Published:** 2018-01-12

**Authors:** Jianmei Wang, Xiaowen Huang, Pei Zhao, Xueying Wang, Ye Tian, Chengmin Chen, Jianchun Wang, Yan Li, Wei Wan, Hanmei Tian, Min Xu, Chengyang Wang, Liqiu Wang

**Affiliations:** 1 School of Chemical Engineering and Technology, Tianjin University, Tianjin 300072, China; wangjm@sderi.cn; 2 Energy Research Institute, Qilu University of Technology (Shandong Academy of Sciences), Jinan 250014, China; huangxiaowen2013@gmail.com (X.H.); zhaop@sderi.cn (P.Z.); wangxy@sderi.cn (X.W.); chencm@sderi.cn (C.C.); wangjc@sderi.cn (J.W.); liyan@sderi.cn (Y.L.); wanw@sderi.cn (W.W.); tianhm@sderi.cn (H.T.); xumin@sderi.cn (M.X.); 3 Department of Mechanical Engineering, The University of Hong Kong, Hong Kong, China; tianye@hku.hk

**Keywords:** microfluidics, monodisperse, microsphere, resol, curing

## Abstract

Monodisperse resorcinol formaldehyde resin (RF) microspheres are an important polymeric material because of their rich surface functional group and uniform structural characteristics and have been increasingly applied as an electrode material, catalyst support, absorbent, and carbon microsphere precursor. The polymerization conditions, such as the gelation/solidification temperature and the residence time, can largely influence the physical properties and the formation of the 3D polymeric network of the RF microspheres as well as the carbon microspheres. However, few studies have reported on the complexity of the gelation and solidification processes of resol. In this work, we developed a new RF microsphere preparation device that contains three units: a droplet generation unit, a curing unit, and a collection unit. In this system, we controlled the gelation and solidification processes of the resol and observed its curing behavior, which helped us to uncover the curing mechanism of resol. Finally, we obtained the optimized polymerization parameters, obtaining uniform RF microspheres with a variation coefficient of 4.94%. The prepared porous RF microspheres presented a high absorption ability, reaching ~90% at 10 min. Thus, our method demonstrated the practicality of on-chip monodisperse microspheres synthesis. The product was useful in drug delivery and adsorbing large poisonous molecules.

## 1. Introduction

Monodisperse spherical resorcinol formaldehyde resin (RF) microspheres are attractive for their rich functional group, uniform size, tunable structure, high carbon content, and relatively low impurity. These advantages make them a good material or carrier in biomedicine, chemical engineering, environmental science, and energy storage [1,2,3,4]. Many efforts have contributed to producing uniform resol resin microspheres, such as hydrothermal synthesis, emulsion polymerization, and spray drying [5,6,7]. Notably, the droplet-based microfluidic technique is useful for the fabrication of microparticles, including hydrogel-based microparticles, plastic-based microparticles, and certain microcapsules [8,9,10,11]. Compared with conventional methods, the droplet-based microfluidic technique can precisely control the entire material fabrication processes, and the microscale size benefits the mass and heat transfer rate [11,12,13,14]. Generally, highly monodisperse emulsified droplets are cured by chemical, photochemical, or physical methods to form uniform microparticles [15]. The droplet curing process largely affects the microparticles’ size, size distribution, and morphology as well as the 3D network of polymer spheres. Unsuitable operation may destroy the monodispersity of the microspheres. For example, magnetic stirring may split the microparticles into fragments with irregular shapes [16]. High-temperature curing may cause demulsification [17] and strongly influence their applications. Thus, research on the curing process can help to develop products meeting various demands arising from applications [18]. 

However, most literature focuses more on the general or early stage reaction mechanism analysis and less on the complexity of the two curing processes (gelation process: A-stage to B-stage; solidification process: B-stage to C-stage) of resol [19,20,21,22]. Therefore, the development of a simple but effective resol curing method is necessary [18,23].

In this study, we developed a glass-chip system including a droplet generation unit, a two-stage curing unit, and a collection unit to synthesize RF microspheres. We monitored the two curing processes via microfluidic technology and obtained uniform spherical RF microspheres. These investigations allowed us to track the network formation process in real time and further understand the curing mechanism and obtain a customized product.

## 2. Materials and Methods 

The resol solution (solid content is 40–42 wt %, viscosity is 12–17 MPa·s) was purchased from Kaihua resin Co., Ltd. (Jinan, China). Urotropine (AR) and liquid paraffine were purchased from Sinopharm Chemical Reagent Co., Ltd. (Shanghai, China). ABIL EM90 was purchased from Degussa Chemical Co., Ltd. (Shanghai, China). A glass capillary tube (inner and outer diameters of 0.58 mm and 1.0 mm, respectively) was purchased from World Precision Instruments (Sarasota, FL, USA), and a square capillary (an inner diameter of 1.05 mm) was purchased from Beijing Chengsheng Apparatus Co., Ltd. (Beijing, China).

**Figure 1:**
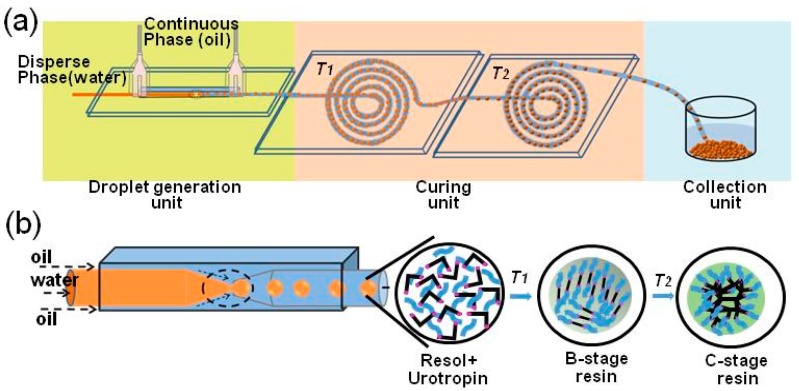
(**a**) Schematic diagram of the formaldehyde resin (RF) microspheres preparation. (**b**) Schematic diagram of the resol inside the droplet during the curing process.

The schematic diagram of the experimental setup was shown as Figure 1a. A typical glass capillary device was used to generate monodisperse water-in-oil (W/O) emulsion. The microfluidic device as per [24] was assembled. The round glass capillary was tapered by a micropipette puller (Sutter P-97, Sutter Instrument Company, Novato, CA, USA). The capillary tip was polished to the desired diameter using sandpaper. The two tapered capillaries were inserted into a square capillary and coaxially aligned. For the generation of W/O emulsions, the surfaces of the circular glass capillary were treated hydrophobically using trimethoxy(octadecyl)silane (technical grade, 90%, purchased from Aldrich chemistry (Sigma-Aldrich Corporation, St. Louis, MO, USA)). The microfluidic curing unit was composed of two layers: the top layer is a glass-based microchannel pattern and the bottom layer is a silicon wafer. The microchannel pattern was designed with AutoCAD software and fabricated via laser engraving. The curing unit was heated by a plane plate heater. 

Both the disperse (a mixture of resol and urotropine) and continuous solutions (liquid paraffin containing 5 wt % ABIL EM90) were separately added into syringes placed on syringe pumps (LSP01-2A, Baoding Longerpump Co., Ltd., Baoding, China), and they were injected at constant flow rates into the device. The W/O droplets were formed at the junction of the microchannel. The droplets were then flushed into the curing unit, in which the gelation and solidification reactions of the resol were performed separately. The resultant microspheres were washed with acetone and alcohol several times to remove the residual oil and the surfactant. 

The emulsion droplets and resultant products were dropped on the glass slide for observation with an inverted optical microscope (T-100, Nikon, Tokyo, Japan). The curing process was monitored in real time by a high-speed camera (Phantom Micro M310, Vision Research Inc., Wayne, NJ, USA). The surface morphology images of the samples were recorded using scanning electron microscopy (SEM, EVOMA10, Carl Zeiss, Oberkochen, Germany). The coated samples were sprayed with gold for 15 s and scanned at 5 kV. The statistical analysis of the microsphere size distribution from the SEM images was performed by ImageJ software. Nearly one hundred particles were analyzed in each picture. The microsphere size distribution was characterized by the coefficient of variation (CV). The microspheres in methylene blue alcohol solution (*w*/*v* = 10 mg/mL) were oscillated at 200 rpm (room temperature), followed by centrifugation. The supernatant was then measured by an ultraviolet-visible (UV-vis) spectrophotometer, and the methylene blue adsorption ratio (*M*) was calculated according to the following:

(1) $$M=\frac{C_{0}-C_{i}}{C_{0}}\times 100\%$$

where *C*_0_ is the initial methylene blue concentration (mmol/L), which equals to 0.05 mM, and *C_i_* is the concentration of methylene blue at time *t_i_* (mmol/L).

## 3. Results and Discussion

### 3.1. Experimental Setup

The schematic diagram of the RF microspheres fabrication system is shown in Figure 1a. It contains three units: a droplet generation unit, a two-stage curing unit, and a collection unit. The tip diameters of the injection and collection capillaries are 120 µm and 250 µm, respectively. The curing unit and the droplet formation unit were separated because a high curing temperature may hinder the normal formation of the droplet. 

The droplet flew into the first curing stage one by one and was subjected to temperature *T*_1_. The temperature of the emulsion droplet rose rapidly to *T*_1_, and the gelation reaction took place at the same time. After that, the droplet kept flowing into the second stage and was subjected to temperature *T*_2_. The temperature of the droplet then rapidly achieved *T*_2_ and solidified. Holding time at each stage was manipulated by the length of the microchannel, the relationship between them was obtained from the following:

(2) $$l=\frac{\left(Q_{c}+Q_{d}\right)\times t}{wh}$$

where *l* is the length of the microchannel (cm), *Q_c_* and *Q_d_* are the flow rate of the continuous phase and the dispersed phase, respectively (mL/min), *t* is the holding time (min), *w* and *h* are the width and the height of the channel (cm). In this study, both the width and the height of the channel are 500 ± 10 μm. To exactly control the curing temperature, the plane plate heater was fixed at each chip bottom, which guaranteed even heating.

### 3.2. Fabrication of the RF Microspheres

A co-flow micro-device was used to prepare the single resol emulsion droplets; the continuous and dispersed fluid flow rate was 0.0167 mL/min and 0.0084 mL/min, respectively. The uniform emulsion droplet with a diameter of 180 ± 5 μm was obtained under this condition. In the first curing unit, the resol inside the droplet began to cross-link and shrink gradually under *T*_1_. The center of the droplet changed from transparent to black. After some time (*t*_1_), the size of the black center kept constant, presenting a gelation reaction (A-stage to B-stage). At *T*_2_, the B-stage resin continued to condense and formed a 3D network structure (B-stage to C-stage). Since the temperature and holding time are two key factors in the curing process, we studied them.

#### 3.2.1. The Effect of the Gelation Temperature and Holding Time

**Figure 2:**
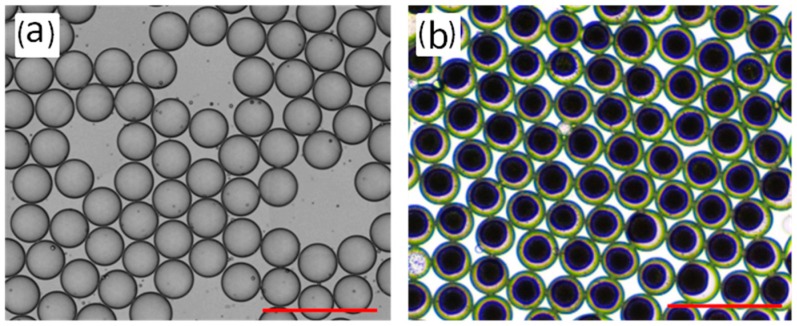
(**a**) Optical microscopic image of the resol emulsion droplet. (**b**) Optical microscopic image of the resol gel inside the droplet, the scale bar is 500 μm.

Resol is a kind of A-stage thermosetting phenolic resin, whose molecular structure has many hydroxyl methyl active groups. A polycondensation reaction between hydroxyl methyl groups made a three-dimensional network [23]. The gelation temperature is crucial in accelerating the crosslinking during chemical reactions. The relationship between gelation time and gelation temperature is shown in Table 1. A higher temperature resulted in faster gel formation. However, in the curing process of the emulsion droplet, when the temperature exceeded the boiling point of the dispersed phase (≥100 °C), droplet demulsification occurred and was deposited at the bottom of the channel. Thus, the best gelation temperature is 90 °C. The optical image of the resol emulsion droplets produced by the co-flow micro-device was shown in Figure 2a, the formed droplets are uniform and transparent. Phase separation occurs inside the droplet during the gelation process (in Figure 2b), which may be caused by the polycondensation reaction between resol molecules. After 20 min, the diameter of the gel inside the droplet slowly changed. Therefore, RF gelled at 90 °C, and this was held for 20 min in the following experiments.

#### 3.2.2. The Effect of the Solidification Temperature 

**Figure 3:**
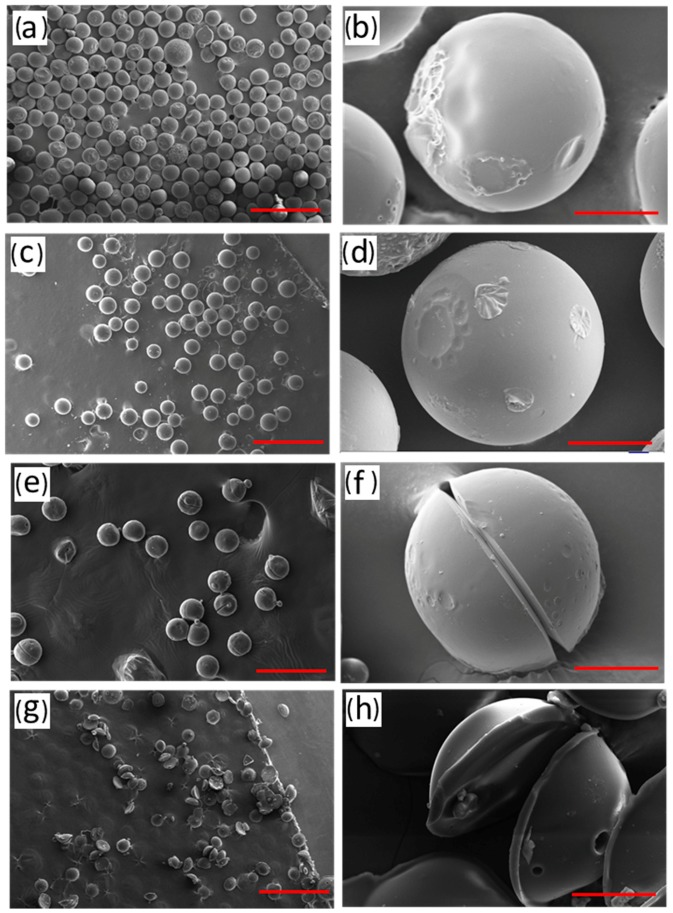
SEM images of RF microspheres with different solidification temperatures: (**a**,**b**) 100 °C; (**c**,**d**) 110 °C; (**e**,**f**) 120 °C; (**g**,**h**) 130 °C; the scale bar for (**a**,**c**,**e**,**g**) and (**b**,**d**,**f**,**h**) is 500 μm and 50 μm, respectively.

The morphology of the RF microspheres is mainly determined by the solidification temperature. The SEM micrographs of RF microspheres with different solidification temperature are shown in Figure 3. Here, the holding time *t*_2_ was fixed to 1 h. All samples presented a uniform sphere shape when *T*_2_ was within the range of 100–120 °C. However, when *T*_2_ exceeded 120 °C, the spheres split in a radial direction (Figure 3c,d).

The above results suggest that, when *T*_2_ was lower than 110 °C, the polycondensation reaction inside the droplet was incomplete and that conglutination occurs between microspheres during washing. When *T*_2_ was higher than 120 °C, the reaction rate rapidly increased. There was not enough time for the steam, produced from the polycondensation reaction and the feedstock, to overflow the microsphere. When energy accumulated to a maximum value, the microsphere split to relieve stress. With the rise in temperature, this phenomenon worsened. Thus, by adjusting the curing temperature *T*_2_, we can effectively control the morphology of the microspheres.

#### 3.2.3. Effect of the Solidification Time

**Figure 4:**
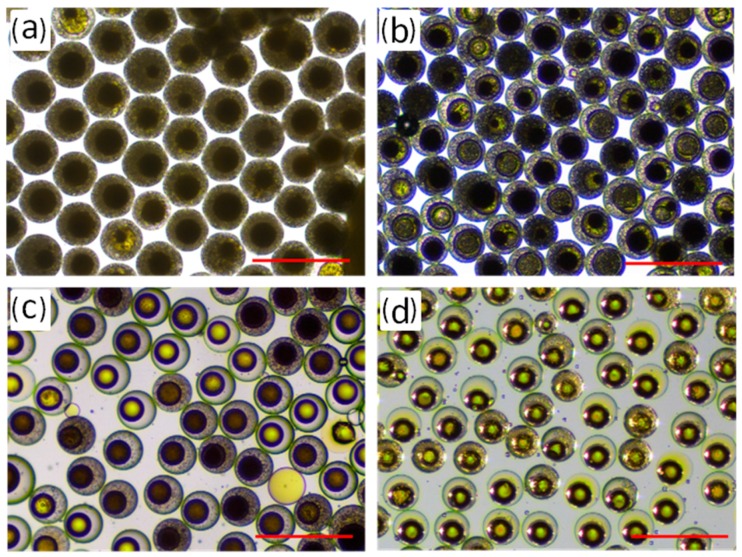
Optical microscopic images of the RF microsphere inside droplet at different holding times: (**a**) 10 min; (**b**) 15 min; (**c**) 20 min; (**d**) 30 min; the scale bar is 500 μm.

The polymerization degree of the RF microspheres largely depends on the holding time (*t*_2_), which is related to the moving distance of the drop in the microchannel. The optical microscopic images of the RF microsphere inside the droplet with different holding times at 110 °C are shown in Figure 4. From Figure 4a, we can see that there are many uniform bubbles generated after the droplet moved from the gelation unit to the solidification unit. These bubbles resulted from the decomposition of urotropine to formaldehyde and ammonia at high temperatures. With the solidification time extension, the color of the compound inside the droplets changed from black to brown (Figure 4b). The bubbles then shrank gradually and finally disappeared (Figure 4c). Finally, the RF microsphere inside the droplet showed luster over time (Figure 4d). Thus, the length of the solidification unit channels was about 120 cm. This tendency can be attributed to the formation of solid particles and structural networks of RF microspheres with a higher degree of polymerization.

**Figure 5:**
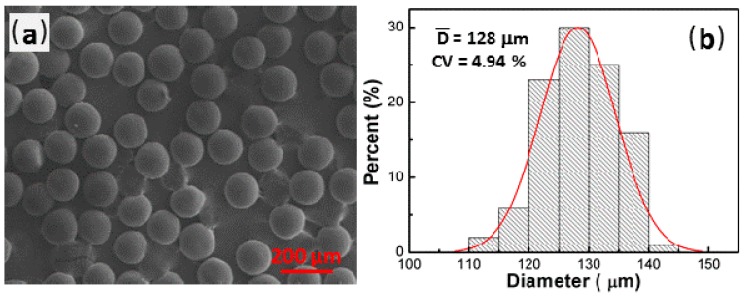
(**a**) Scanning electron microscopy (SEM) micrographs of RF microspheres; (**b**) particle size distribution of microspheres. Curing conditions: *T*_1_ = 90 °C, *t*_1_ = 20 min; *T*_2_ = 110 °C, *t*_2_ = 30 min.

The SEM micrographs and the particle diameter distribution of RF microspheres synthesized under optimum curing conditions are shown in Figure 5. From this curing method, we obtained microspheres with an average particle size ($\bar{D}$) of 128 μm, excellent sphericity and a narrow size distribution (CV = 4.94%). 

#### 3.2.4. Adsorption Test of the Porous RF Microspheres

**Figure 6:**
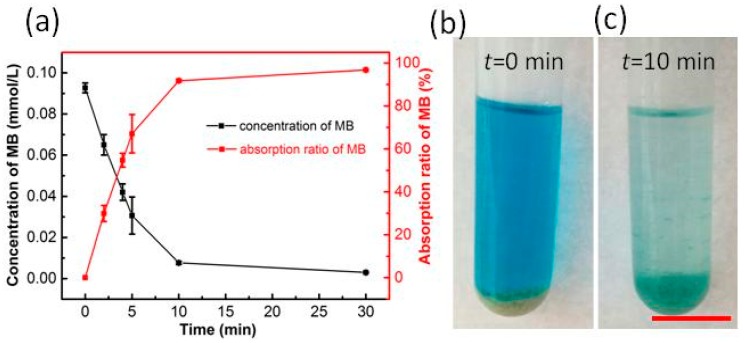
Adsorption test of the porous RF microspheres; (**a**) adsorption curve of methylene blue; (**b**) before adsorption photos; (**c**) after 10 min adsorption. The scale bar is 1.0 cm.

Porous RF microspheres were prepared by the mentioned process with the 0.5% polyvinylpyrrolidone (*w/w*) in the resol solvent. To test the adsorption ability of the porous RF microspheres, the methylene blue (MB) alcohol solution was taken as the adsorption model. As shown in Figure 6, the methylene blue adsorption reached ~90% at 10 min, showing a high adsorption ability, which may be useful as absorbents for adsorbing large poisonous molecules, drug delivery, and pollution removal [25,26].

## 4. Conclusions

We have synthesized monodispersed RF microspheres and visualized the resol resin curing process via microfluidic technology. The two-stage curing unit can effectively distinguish the gelation and solidification processes for resol curing. RF microspheres with good sphericity and uniform size were prepared based on this curing system. The temperature and holding time of each process significantly affected the resol curing degree and RF microsphere morphology. The curing process was implemented sequentially via gelation and solidification processes, and elevated curing was achieved for the resol inside the droplet. This method is novel in terms of reactions inside the droplet, particularly multistep reactions. This technique is expected to be applicable to not only the preparation of uniform RF microspheres but also the study of microchannel reaction mechanism combining with other online analytical tools.

## Figures and Tables

**Table 1:** The relationship between gelation time and gelation temperature.

***T*_1_ (°C)**	***t*_1_ (min)**	***L* (cm)**
80	>60	239
90	20	79
100	not detected	not detected
